# Obesity: should there be a law against it? Introduction to a symposium

**DOI:** 10.1186/1743-8462-5-9

**Published:** 2008-06-05

**Authors:** Roger S Magnusson

**Affiliations:** 1 Faculty of Law, University of Sydney, Sydney, Australia

## Abstract

The rapid rise in rates of overweight and obesity among adults and children in Australia and New Zealand has intensified debate about the most effective policies for obesity prevention. Law has much to contribute to this policy discussion, although its role is often misunderstood. The articles in this symposium follow on from a conference hosted in September 2006 by the Centre for Health Governance, Law & Ethics in the Faculty of Law, University of Sydney, titled: *Obesity: should there be a law against it? *In different ways, these articles provide a variety of perspectives on regulatory responses to obesity, including theoretical justifications for a legal approach, conceptual models that assist in making sense of law's role, as well as specific legal strategies for obesity prevention in various settings.

## Editorial

What can law do about obesity? Law's role in preventing and reversing weight gain at the population level has become a serious topic of discussion among public health lawyers and policy-makers. In the United States, there has been tremendous interest in the extent to which food and beverage companies could be vulnerable to tobacco-style lawsuits brought by obese claimants for the health effects of obesity and chronic diseases [1-5]. Legal interest has broadened as lawyers have begun to consider the role that law might play as a policy tool in obesity prevention efforts. Law's capacity to address childhood obesity, including school-based interventions, and the regulation of advertising, has emerged as an important theme in the literature [6-8]. Parliamentary inquiries – most recently in New Zealand – have proposed policies for the future [9,10]. Legislatures, meanwhile, at least in the United States, have not stood still, and recent reviews (including Hodge, Garcia and Shaw in this symposium) demonstrate the wide range of laws that have already been introduced as part of obesity prevention efforts [11-13].

Public health scholars have long recognized, although sometimes implicitly, a role for law in policy approaches to obesity and chronic diseases generally [14-20]. Growing interest in these issues among public health lawyers themselves is therefore timely and appropriate.

## Obesity – should there be a law against it?

The articles in this symposium aim to open up the debate about law and obesity for lawyers and non-lawyers alike, with specific reference to Australia, but informed by experience in the United States and Britain. Early versions of some of the articles in this series were originally presented at a conference hosted by the University of Sydney Faculty of Law in September 2006, provocatively entitled: *Obesity: should there be a law against it? *This conference elicited a strong reaction, with some people writing in from around the world to condemn the conference as yet more evidence of discrimination against obese people. Ironically, it is those who are most opposed to a role for law in the regulation of obesity and chronic disease who have raised (facetiously) the prospect of policies that would tax citizens for their extra kilos [21], or frankly advocated ramping up health insurance premiums for the overweight in order to eliminate the subsidies that thin people pay overweight people through community rated schemes [22].

By extending their gaze beyond the proximate, behavioural determinants of obesity, policy-makers who adopt a population health perspective have a far broader range of policies to choose from. By acknowledging the reality of socioeconomic and environmental influences upon patterns of eating and physical activity, a population health perspective is more sensitive to the challenges that individuals face, and is more likely to avoid legal approaches that are punitive and discriminatory.

## Law and obesity: three broad themes

The title for this symposium raises a critical issue: what does it mean for law to act against obesity? To make sense of this question, and the kinds of answers it elicits, it is helpful to keep three broad themes in mind.

The first theme is the ethical and philosophical justification for using *law *to influence the determinants of obesity. Law is a controversial player in the field of non-communicable diseases. Legal strategies for responding to health threats within liberal societies are least controversial when they focus on pathogens and toxins and "external threats" that are either infectious (like drug-resistant tuberculosis, SARS, or bird flu) or which create the risk of sudden and catastrophic harm to society at large (like bioterrorism). Obesity, on the other hand, literally embodies the daily choices that individuals make, and invites the response that people should be left alone to live their private lives as they see fit. Law needs to justify its role in the shadow of pervasive assumptions about non-interference with individual preferences – and the accompanying ethic of personal responsibility for choices made – that characterizes the liberal state [23-25].

The second theme relates to how, in a conceptual sense, law fits into a public health framework for obesity prevention. Discussion of law and obesity, like law's role in tobacco control, tends to gravitate towards specific, "hot button" issues. But how can we understand the possibilities of law as a policy tool within a broader framework that links these legal strategies with the determinants of obesity?

An effective response to population weight gain begins, but does not end, with clear strategies. It is also critically important to sell policy ideas effectively in the realm of politics. There is an important literature about the factors that prompt political action on public health issues, including obesity, and the importance of framing policy ideas effectively [26,27].

The third theme relates to the detail of specific laws that seek to respond to obesity. While the detail will vary between different countries, and jurisdictions, obesity prevention is a shared challenge. Experimentation with legal and regulatory approaches to obesity prevention is likely to increase, and there is a great deal to learn from a comparative approach.

The articles in this symposium engage in different ways with each of these three themes. **A/Professor James Hodge**, from the Johns Hopkins Bloomberg School of Public Health, and co-authors **Andrea Garcia **and **Supriya Shah**, open the symposium with a review of legal strategies concerning obesity in the United States [13]. **Professor Robyn Martin**, from the University of Hertfordshire, and a visiting Professor at the Chinese University of Hong Kong, writes about what is unique to the food and eating culture of the United Kingdom, and the law's evolving role in obesity policy in England [28].

**Dr Gary Sacks**, and co-authors **Mark Lawrence **and **Boyd Swinburn **from the School of Exercise and Nutrition Science at Deakin University present a conceptual framework for systematically locating the roles of local, state and Commonwealth government in policies across the food system and physical activity environments [29]. In a separate article, **Professor Boyd Swinburn**, from the World Health Organization's Collaborating Centre for Obesity Prevention at Deakin University, identifies law and regulation as one of several broad areas in which leadership from governments is required. In addition to redressing factors that contribute to obesogenic environments, and supporting obesity prevention, there are opportunities for legal and policy efforts to work synergistically with other "movements" for policy action [30].

Drawing on strategies used in environmental regulation, **Professor Stephen Sugarman**, from the University of California, Berkeley, and **Nirit Sandman**, present a novel approach to regulating food manufacturers in order to achieve reductions in childhood obesity [31]. **A/Professor Elizabeth Handsley**, and co-authors **Kaye Mehta, John Coveney and Chris Nehmy**, from Flinders University, evaluate the criteria that could be used for regulating food advertising to children on television [32]. Their analysis is central to the design of effective regulation of children's food advertising, an issue which is gaining momentum in Australia and beyond.

In part 1 of a two-part article, **A/Professor Roger Magnuson**, from the Faculty of Law, University of Sydney, provides a model for understanding the role of law in preventing population weight gain [33]. Part 2 provides a systematic review of possible interventions for law in obesity prevention [34].

How law can best contribute to reducing the health burden of obesity and other lifestyle risk factors for the chronic diseases that Australians overwhelmingly get sick and die from remains one of the most profound challenges that public health law faces in the twenty-first century. The articles in this symposium are by no means an exhaustive review. Law's proper role will continue to be discovered, debated and refined in coming decades.
